# Supplementary material for: Interaction with Nature Indoor: Psychological Impacts of Houseplants Care Behaviour on Mental Well-Being and Mindfulness in Chinese Adults
Source: Int J Environ Res Public Health. 2022 Nov 28;19(23):15810. doi: 10.3390/ijerph192315810 (PMC9739745; doi:10.3390/ijerph192315810)
Supplement: Supplementary file 1 [file ijerph-19-15810-s001.zip › ijerph-2009388-supplementary.pdf]

Section S1. Diagram of Sampling Process

* 22 provinces, 5 autonomous regions, 4 province-level municipalities (excluding Hong Kong, Macau and Taiwan).

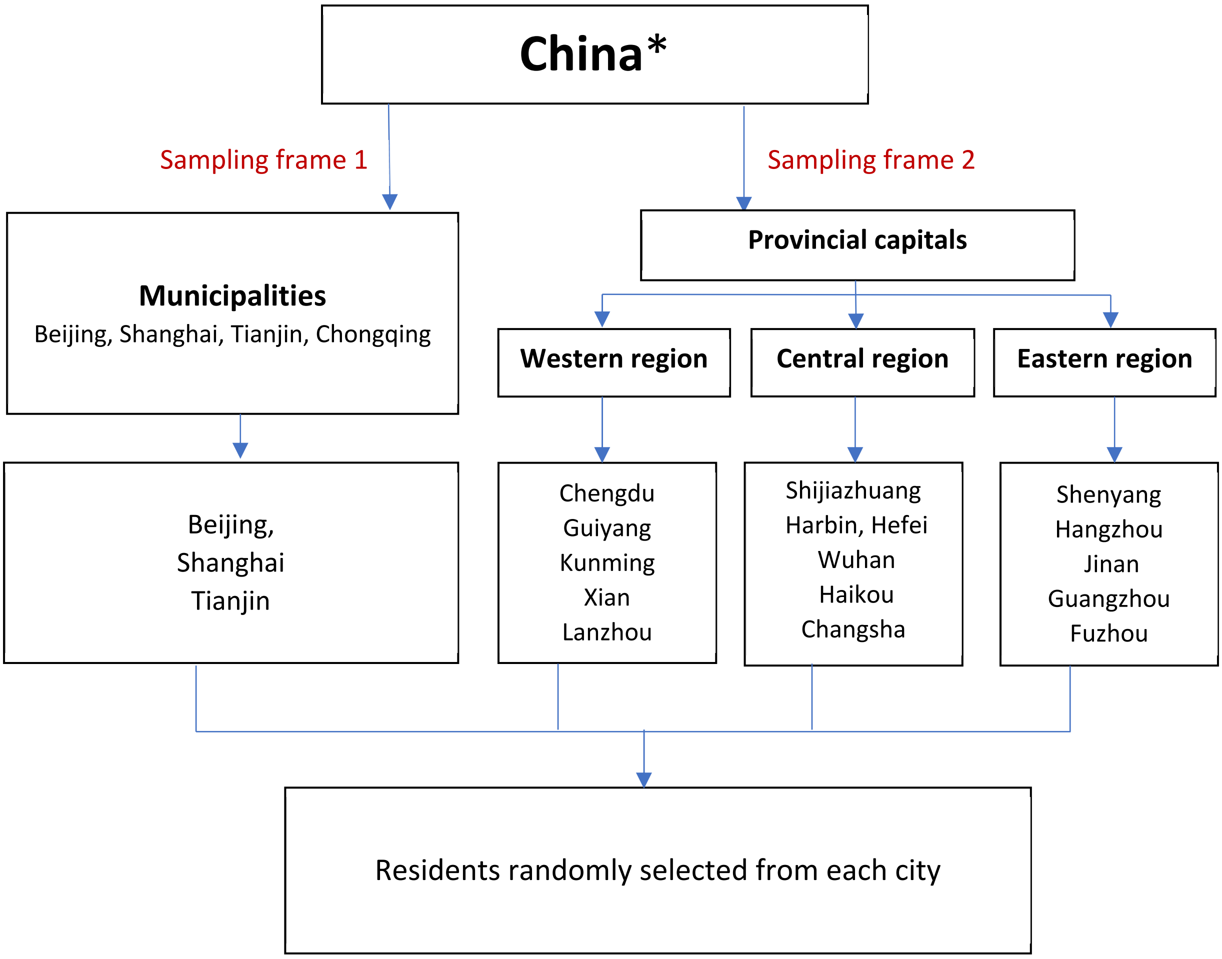

Section S2. Questions of Houseplants Care Behaviour Stage

Which stage are you in for houseplants care (e.g., watering, feeding, looking after, maintaining and repotting, etc.)?

- *Precontemplation*: I'm not ready to do this;
- *Contemplation*: I plan to do this soon;
- *Preparation*: I'm ready to do this in the next 6 months;
- *Action*: I've been doing this within the past 6 months;
- *Maintenance*: I've been doing this for over 6 months;

How long did you spend on home-planting in the last week? ___hour(s)

How many pots of plants do you responsible for looking after at home? ___

For how long have you been planting in your resident(s)? ___year(s)

Section S3. Questions about Preferable Characteristics of Plants

Which **characteristic** of plants are most important to people for planting?

1) Nice scent of plants:

- Extremely unimportant
- Unimportant
- Neither unimportant nor important
- Important
- Extremely important

2) Ease of growing:

- Extremely unimportant
- Unimportant
- Neither unimportant nor important
- Important
- Extremely important

3) Cheaper cost of purchasing and maintaining plants:

- Extremely unimportant
- Unimportant
- Neither unimportant nor important
- Important
- Extremely important

4) Texture of plants (e.g., soft or fragile texture):

- Extremely unimportant
- Unimportant

- Neither unimportant nor important
- Important
- Extremely important

5) Small size of plants:

- Extremely unimportant
- Unimportant
- Neither unimportant nor important
- Important
- Extremely important

6) Green colored plants:

- Extremely unimportant
- Unimportant
- Neither unimportant nor important
- Important
- Extremely important

7) Good looking and ornamental:

- Extremely unimportant
- Unimportant
- Neither unimportant nor important
- Important
- Extremely important
